# Byssinosis and lung health among cotton textile workers: baseline findings of the MultiTex trial in Karachi, Pakistan

**DOI:** 10.1136/oemed-2022-108533

**Published:** 2023-01-30

**Authors:** Asaad Ahmed Nafees, Muhammad Zia Muneer, Muhammad Irfan, Muhammad Masood Kadir, Sean Semple, Sara De Matteis, Peter Burney, Paul Cullinan

**Affiliations:** 1 Department of Community Health Sciences, Aga Khan University, Karachi, Pakistan; 2 National Heart & Lung Institute, Imperial College London, London, UK; 3 Department of Pulmonary and Critical Care Medicine, Aga Khan University, Karachi, Pakistan; 4 Institute of Social Marketing, University of Stirling, Stirling, UK; 5 Department of Medical Sciences and Public Health, University of Cagliari, Cagliari, Italy

**Keywords:** Epidemiology, Occupational Health, Dust, Particulate Matter, Respiratory Function Tests

## Abstract

**Objectives:**

To assess the association of exposure in cotton mills in Karachi with different definitions of byssinosis and lung health.

**Methods:**

This cross-sectional survey took place between June 2019 and October 2020 among 2031 workers across 38 spinning and weaving mills in Karachi. Data collection involved questionnaire-based interviews, spirometry and measurements of personal exposure to inhalable dust. Byssinosis was defined using both WHO symptoms-based (work-related chest tightness), and Schilling’s criteria (symptoms with decreased forced expiratory volume in 1 s (FEV_1_). Values of FEV_1_/forced vital capacity ratio below the lower limit of normality on postbronchodilator test were considered as ‘chronic airflow obstruction’ (CAO).

**Results:**

56% of participants had at least one respiratory symptom, while 43% had shortness of breath (grade 1). Prevalence of byssinosis according to WHO criteria was 3%, it was 4% according to Schilling’s criteria, and likewise for CAO. We found low inhalable dust exposures (geometric mean: 610 µg/m^3^). Cigarette smoking (≥3.5 pack-years), increasing duration of employment in the textile industry and work in the spinning section were important factors found to be associated with several respiratory outcomes.

**Conclusion:**

We found a high prevalence of respiratory symptoms but a low prevalence of byssinosis. Most respiratory outcomes were associated with duration of employment in textile industry. We have discussed the challenges faced in using current, standard guidelines for identifying byssinosis.

**WHAT IS ALREADY KNOWN ON THIS TOPIC**

- Cotton dust exposure among textile workers is known to cause byssinosis—a chronic respiratory condition characterised by the presence of symptoms of chest tightness on return to work after holidays, which may progress to obstructed lung function.
- The disease is common across low-income and middle-income countries.
- Two separate criteria are currently in use for assessing byssinosis—that proposed by the WHO (based on work-related symptoms of chest tightness), and that proposed by Schilling (symptoms with a decline in forced expiratory volume in 1 s (FEV_1_).

**WHAT THIS STUDY ADDS**

- We found a high prevalence of respiratory symptoms; prevalence of byssinosis using the WHO criteria was 3% and using the Schilling’s criteria was 4%.
- Prevalence of chronic airflow obstruction was 4% and that of bronchodilator reversibility in FEV_1_ (≥200 mL) was 15%.
- Increasing duration of employment in the textile industry was associated with increased chances of adverse respiratory outcomes signifying the role of long-term exposure to cotton dust in causing byssinosis.
- We discuss challenges in assessing byssinosis using the current, standard definitions and suggest use of locally validated questionnaires in future studies.

**HOW THIS STUDY MIGHT AFFECT RESEARCH, PRACTICE OR POLICY**

- In this article, we discuss the need to deliberate modified criteria for measuring byssinosis in workforce studies, using a combination of the work-related chest tightness and FEV_1_/forced vital capacity ratio.

## Introduction

Textile manufacture is a major contributor to economies across low-income and middle-income countries (LMICs). Cotton dust exposure among textile workers is known to cause byssinosis—a chronic respiratory condition characterised by the presence of symptoms of chest tightness on return to work after holidays, and progressively leading to obstructed lung function. The disease attracts little attention in high-income countries because improvements in health and safety standards there have resulted in lower dust exposures and disease prevalence.^1 2^ Moreover, much textile manufacture from high-income countries has been relocated to LMICs.

In a recent systematic review, we highlighted that byssinosis remains an important concern across textile processing LMICs.^3^ The review exposed the challenges in determining the burden of disease due to variations in epidemiological approaches to workforce studies and in the use of classification criteria. Currently, two classification systems are followed, that initiated by Schilling *et al* 4 and the subsequent, related WHO system.^5^ Both were developed decades ago, largely in response to studies of cotton textile workers in the UK and USA, and they may need to be adjusted to reflect the current geographic distribution of textile manufacture and the obstacles to the use of such approaches in these countries.

Here, we report findings from the baseline survey of the MultiTex RCT (randomised controlled trial) study^6^ in Karachi, Pakistan, in which we assessed the association of exposure in cotton mills with different definitions of byssinosis and lung health. We describe challenges in the use of current classification systems for assessing byssinosis and suggest recommendations for future studies.

## Methods

### Study design, setting and recruitment

This cross-sectional survey took place between June 2019 and October 2020 in 38 spinning and weaving mills in Karachi with a pause in data collection between March and June 2020 due to the COVID-19 pandemic. We identified a total of 225 spinning and weaving mills in the city and approached 83 to recruit the required number of mills for the study.^6^ Of the 83, 21 did not meet our eligibility criteria,^6^ 16 declined to participate and no response was received from 8. From the remaining 38 mills, 2031 workers were enrolled according to the criteria set out in the trial protocol^6^; in consultation with the mill management, we took a convenience sample of up to 70 workers at each mill, depending on the size of the workforce, who fulfilled the following criteria: (1) age ≥18 years; (2) from spinning or weaving sections (workers from only one section were enrolled from each mill); (3) non-managerial (see below for a list of job titles that were considered); (4) employed in the textile industry for at least 6 months and (5) at work at the time of survey. Of those eligible, and invited to take part, all but five did so. The size of the total workforce at enrolled mills ranged from 26 to 2600.

### Interviews

Trained data collectors conducted questionnaire-based interviews, entering real time data using an android application, Epicollect5.^7^ The study questionnaire has been adopted from the MRC and WHO respiratory questionnaires^8–10^; these questions have been previously used in several studies among Pakistani textile workers^11^ and have been locally validated.^12^ The questions on wheezing have been shown to have good validity using bronchial response to histamine across diverse ethnolinguistic groups.^13^ The questionnaire was translated into Urdu and back translated in English and pilot tested prior to use in the trial. ‘Chronic’ cough and phlegm were defined by the presence of these symptoms for at least three consecutive months a year, for at least 2 years. Those reporting both chronic cough and chronic phlegm were categorised as having ‘chronic bronchitis’. Wheezing was defined by whistling sounds from the chest during the last 12 months. Breathlessness was categorised into grades 1, 2 and 3, in increasing order of severity.^8^ A ‘composite’ respiratory variable was defined by the presence of one or more of the above respiratory symptoms and chest ‘tightness’.

We considered two separate definitions of byssinosis—according to Schilling *et al*’s criteria,^4^ byssinosis was categorised based on the presence of chest tightness on return to work after ‘regular scheduled weekly holidays’, or vacations, into: grade ½ (symptoms on some of the first days back at work); grade 1 (most of the first days back at work); grade 2 (first and other days) and grade 3 (first and other days and forced expiratory volume in 1 s (FEV_1_) percentage-predicted value <80%). WHO symptoms-based ‘byssinosis’^5^ was defined in a similar way, using presence of chest tightness, categorised into: B1 (symptoms on most of the first days back at work) and B2 (on both first and other days of the workweek).

### Spirometry and anthropometric measurements

We recorded height in cm using a stadiometer. Trained technicians used the EasyOne (ndd Medizintechnik) spirometer to measure prebronchodilator and postbronchodilator (two puffs of salbutamol 100 µg)^14^ lung function values, following established guidelines,^15^ including FEV_1_ and forced vital capacity (FVC) in L, and their ratio (FEV_1_/FVC) in percentage. Spirometry was performed within respective textile mills during working hours. An improvement in FEV_1_ of ≥200 mL on postbronchodilator testing was considered as evidence of bronchodilator ‘response’ (BDR). We defined chronic airflow obstruction (CAO) as a postbronchodilator FEV_1_/FVC value below the lower limit of normality (LLN), derived from the third US National Health and Nutrition Examination Survey (NHANES-III; ‘Caucasian’) reference equations.^16^

Spirometry was undertaken on 1747 (86%) workers. During the COVID-19 pandemic, 216 workers were interviewed without spirometry; 6 employees had a contraindication to spirometry, 1 declined it and 61 were unable to perform the test. After the exclusion of a further 26 men who could not produce measurements of acceptable quality, a total of 1723 workers were included in the analyses of spirometry; they produced 1721 useable prebronchodilator and 1712 postbronchodilator measurements.

### Cotton dust sampling

We used IOM sampling heads with glass fibre filters and Casella Apex2 pumps to measure personal exposures to the inhalable fraction of particulate matter (PM<100 µm) over a shift following standard guidelines.^17^ We weighted filters in a temperature and humidity-controlled environment; changes in weights were recorded in micrograms. We calculated the 8-hour time-weighted average concentrations of inhalable dust^18^ at each mill; sampling was undertaken on purposively selected workers categorised into four groups: (1) helpers, cleaners and doffers; (2) machine operators; (3) technicians, jobbers and fitters; and (4) masters, chargehands or supervisors.

We took personal air samples for 184 workers (9% of all participants) from 37 mills (15 spinning and 22 weaving), excepting just 1 mill where sampling could not be undertaken due to difficulty in obtaining access. We discarded 16 samples due to an apparent decrease in the postsampling weight of filters. Among the remaining, the overall median (IQR) sampling duration was 6^5–7^ hours. With respect to job title, the largest number of samples were taken from machine operators (42%; n=71) and the fewest from cleaners (22%; n=37).

### Statistical analysis

All stastistical analyses were undertaken using STATA (V.15). Since age and duration of employment in the textile industry were not normally distributed, we converted them into categorical variables using the cut-off values for their quartiles (Q1–Q4). The high collinearity between these variables when using the original (continuous) information (Pearson’s r=0.72) was largely eliminated after categorisation (Cramer’s V=0.43). Similarly, we did not find high collinearity between categories of age and of pack-years of smoking (Cramer’s V=0.22).

Based on a priori considerations, we assessed associations between several personal and work-related covariables and respiratory health outcomes using logistic and linear multivariable regression models. The covariables included sociodemographic measures (age, income, education), body mass index and occupational (duration of employment in textile industry, job title, working hours, nature of job contract), mill-related (section, type and size of mill) and smoking (pack-years of smoking) variables.

We initially examined the associations in univariable models. Covariables with p<0.25 and those with strong biological plausibility were further assessed in multivariable models. We used a backward stepwise regression approach to model building and report adjusted coefficients and ORs with 95% CIs for the final models.

## Results

Among the 38 participating mills, the majority were weaving mills (61%; n=23) and among these, three different types of looms were installed; in increasing order of technological advancement, power, shuttle-less and air-jet looms, shuttle-less being the most common (44% of mills; n=10). The spinning mills consisted entirely of the ‘ring’ type—that uses a rather more traditional approach for spinning yarn compared with the alternate newer approach of ‘open-end’. The ‘open-end’ type has reduced steps in yarn spinning thus supposedly increasing the overall productivity and resulting in comparatively less dust exposure.^19^ We used the Pakistan Cotton Standards Institute grading criteria to categorise the spinning mills into two groups, based on the quality of cotton they used as raw material. ‘Non-lint content’, is a term used to grade the quality of cotton and represents the proportion of impurities such as, particles of sand, leaves, which might be present. We considered mills using cotton with non-lint content up to 3% as ‘high grade’; and those with more than 3% as ‘low grade’^20^; and in our sample found most using ‘high grade’ cotton (60%; n=9). Most of the mills included in this study were using natural cotton fibre.

The mean (±SD) age of participants was 31 (±9.5) years, and their median duration of work in the textile industry was nine (IQR: 4–16) years. Around half of the employees reported no schooling. The prevalence of cigarette smoking (‘ever in lifetime’) was 24% (n=487); among those who had ever smoked, the accumulated quantity of cigarettes smoked was low, with a median of 3.5 pack-years. Previous diagnoses of pneumonia, tuberculosis or self-reported asthma were rare (each less than 3%). Workers were almost equally divided across the two types of mills, spinning and weaving; three quarters reported their job status to be ‘permanent’ while the rest were ‘on contract’. The most common job title was ‘machine operator’ (58%; n=1183), followed by ‘cleaner’ (22%; n=436).

Shortness of breath (grade 1) was the most common respiratory symptom (43%; n=873), with 7% (n=151) reporting the most severe category (grade 3). Chronic cough was reported by 13% (n=267), chronic phlegm by 15% (n=301) and chronic bronchitis by 8% (n=158); 15% (n=307) of employees reported ‘current’ chest tightness. The prevalence of the composite variable for respiratory symptoms was 56% (1145 workers). Using WHO symptoms-based criteria, the prevalence of any degree of byssinosis was 3% (n=60; 95% CI 2% to 4%); according to Schilling’s criteria the equivalent figure was 4% (n=67; 95% CI 3% to 5%). Approximately 2% (n=32) of participants had the most severe grade of byssinosis according to the latter classification.

In the adjusted models (table 1), compared with non-smokers, those reporting 3.5 or more pack-years of smoking were more likely to report chronic cough and phlegm, and the composite variable. Across the gradient of pack-years, the adjusted ORs increased monotonically for reports of chronic phlegm and for the composite respiratory variable. After adjustment for the variables above, and for age, an association between the composite respiratory outcome and duration of employment in the textile industry remained statistically significant, with increasing ORs across the quartiles of duration. Compared with weavers, those working in spinning sections were less likely to report the composite respiratory variable (AOR 0.72; 95% CI 0.59 to 0.89).

**Table 1:** Univariable and multivariable logistic regression analyses of individual and workplace factors in association with respiratory symptoms and byssinosis (n=2031)

Variables	n (%)	Chronic cough (n=267; 13%)		Chronic bronchitis* (n=158; 8%)		Chest tightness, current (n=307; 15%)		Composite respiratory variable† (n=1145; 56%)		Byssinosis‡ (n=60; 3%)	
		OR (95% CI)	AOR (95% CI)	OR (95% CI)	AOR (95% CI)	OR (95% CI)	AOR (95% CI)	OR (95% CI)	AOR (95% CI)	OR (95% CI)	AOR (95% CI)
Age (years)§	30 (24–37)¶										
Q1 (18–24)	–	1.00	1.00	1.00	1.00	1.00	1.00	1.00	1.00	1.00	–
Q2 (25–30)	–	1.12 (0.77 to 1.61)	0.95 (0.64 to 1.40)	1.4 (0.88 to 2.27)	1.12 (0.66 to 1.89)	0.96 (0.69 to 1.35)	0.77 (0.53 to 1.12)	1.07 (0.84 to 1.35)	0.83 (0.63 to 1.09)	1.30 (0.64 to 2.63)	–
Q3 (31–37)	–	1.51 (1.0 to 2.16)	0.95 (0.61 to 1.48)	1.78 (1.11 to 2.85)	0.87 (0.48 to 1.57)	1.40 (1.01 to 1.94)	0.84 (0.55 to 1.27)	1.07 (0.84 to 1.35)	0.61 (0.44 to 0.84)	1.41 (0.69 to 2.88)	–
Q4 (38–71)	–	1.34 (0.93 to 1.93)	0.69 (0.42 to 1.13)	1.63 (1.01 to 2.61)	0.56 (0.28 to 1.10)	0.80 (0.56 to 1.14)	0.38 (0.23 to 0.62)	1.25 (0.98 to 1.60)	0.53 (0.37 to 0.76)	0.99 (0.46 to 2.13)	–
BMI**											
Normal	892 (49.2)	1.00	–	1.00	–	1.00	1.00	1.00	1.00	1.00	–
Underweight (<18.5)	269 (14.8)	1.24 (0.85 to 1.81)	–	0.99 (0.60 to 1.65)	–	0.96 (0.67 to 1.40)	0.92 (0.63 to 1.34)	1.21 (0.92 to 1.61)	1.13 (0.86 to 1.52)	1.24 (0.59 to 2.59)	–
Overweight and obese (>23.9)	654 (36.0)	1.14 (0.86 to 1.52)	–	1.23 (0.86 to 1.76)	–	0.98 (0.74 to 1.28)	1.00 (0.74 to 1.34)	1.35 (1.10 to 1.67)	1.32 (1.05 to 1.66)	1.12 (0.63 to 1.98)	–
Pack-years of smoking											
None	1544 (76.0)	1.00	1.00	1.00	1.00	1.00	1.00	1.00	1.00	1.00	1.00
<3.5	239 (11.8)	1.93 (1.35 to 2.76)	1.82 (1.27 to 2.63)	2.29 (1.48 to 3.55)	2.25 (1.43 to 3.53)	1.55 (1.09 to 2.20)	1.43 (0.99 to 2.07)	1.93 (1.45 to 2.58)	1.77 (1.31 to 2.41)	2.53 (1.35 to 4.76)	2.47 (1.30 to 4.67)
≥3.5	248 (12.2)	2.09 (1.48 to 2.96)	1.86 (1.28 to 2.71)	2.80 (1.86 to 4.21)	2.26 (1.43 to 3.59)	1.56 (1.10 to 2.20)	1.50 (1.03 to 2.21)	2.87 (2.12 to 3.90)	2.78 (1.96 to 3.93)	1.53 (0.73 to 3.22)	1.37 (0.63 to 2.99)
Duration of employment in the textile industry (years)§	9.0 (4.0–16.0)¶										
Q1 (0–4)	–	1.00	1.00	1.00	1.00	1.00	1.00	1.00	1.00	1.00	1.00
Q2 (5–9)	–	1.30 (0.88 to 1.94)	1.32 (0.86 to 2.03)	1.86 (1.08 to 3.20)	2.00 (1.09 to 3.65)	1.23 (0.86 to 1.77)	1.28 (0.85 to 1.92)	1.42 (1.11 to 1.82)	1.49 (1.11 to 1.98)	0.84 (0.36 to 2.13)	0.82 (0.35 to 1.95)
Q3 (10–16)	–	1.48 (1.0 to 2.17)	1.51 (0.96 to 2.39)	2.07 (1.22 to 3.52)	2.19 (1.15 to 4.18)	1.54 (1.09 to 2.17)	1.69 (1.09 to 2.61)	1.70 (1.33 to 2.16)	1.79 (1.30 to 2.47)	1.59 (0.78 to 3.22)	1.51 (0.70 to 3.27)
Q4 (17–46)	–	2.14 (1.48 to 3.11)	2.53 (1.48 to 4.33)	3.24 (1.95 to 5.39)	4.12 (1.96 to 8.65)	1.57 (1.10 to 2.23)	2.53 (1.49 to 4.29)	1.99 (1.55 to 2.57)	2.26 (1.52 to 3.35)	1.52 (0.73 to 3.16)	1.48 (0.63 to 3.45)
Section of mill											
Weaving	983 (48.4)	1.00	–	1.00	1.00	1.00	–	1.00	–	1.00	1.00
Spinning	1048 (51.6)	0.97 (0.75 to 1.25)	–	1.13 (0.82 to 1.57)	1.38 (0.97 to 1.95)	1.01 (0.79 to 1.29)	–	0.81 (0.68 to 0.96)	–	1.07 (0.64 to 1.80)	1.2 (0.71 to 2.03)
Job title											
Supervisor	154 (7.6)	1.00	1.00	1.00	1.00	1.00	1.00	1.00	1.00	1.00	1.00
Technician	258 (12.7)	1.11 (0.60 to 2.04)	1.40 (0.75 to 2.63)	0.99 (0.47 to 2.10)	1.64 (0.73 to 3.68)	1.08 (0.62 to 1.87)	1.24 (0.68 to 2.27)	1.03 (0.69 to 1.55)	1.23 (0.77 to 1.96)	0.89 (0.25 to 3.22)	1.04 (0.29 to 3.82)
Machine operator	1183 (58.2)	1.24 (0.74 to 2.08)	1.75 (1.01 to 3.00)	1.09 (0.59 to 2.04)	1.90 (0.93 to 3.86)	1.06 (0.66 to 1.69)	1.23 (0.74 to 2.07)	0.90 (0.64 to 1.27)	1.09 (0.73 to 1.61)	1.28 (0.45 to 3.63)	1.58 (0.54 to 4.61)
Cleaner	436 (21.5)	0.96 (0.54 to 1.70)	1.80 (0.95 to 3.39)	0.75 (0.37 to 1.53)	2.16 (0.93 to 4.99)	0.87 (0.52 to 1.47)	1.44 (0.79 to 2.64)	0.68 (0.47 to 0.99)	1.17 (0.74 to 1.85)	0.97 (0.30 to 3.10)	1.48 (0.42 to 5.21)
Working hours											
≤8	1298 (63.9)	1.00	1.00	1.00	–	1.00	–	1.00	–	1.00	–
>8	733 (36.1)	0.74 (0.56 to 0.98)	0.70 (0.52 to 0.93)	0.76 (0.53 to 1.08)	–	0.86 (0.67 to 1.11)	–	0.77 (0.64 to 0.92)	–	0.82 (0.47 to 1.42)	–

All variables in the multivariable models are mutually adjusted; blank cells represent covariables that were dropped in the process of stepwise model building

*Those having both cough and phlegm for 2 years or more.

†Includes participants reporting one or more of respiratory symptoms (chronic cough, chronic phlegm, increased cough and phlegm during last 3 years, wheezing, breathlessness grades 1, 2 or 3, and chest tightness).

‡WHO symptoms-based criteria.

§Continuous variables were converted into quartiles; Q1–Q4 correspond to the cut-off values for each quartile.

¶Median (IQR) reported.

**Data available for 34 mills (n=1815).

AOR, adjusted OR; BMI, body mass index.

In multivariable models for the outcome ‘byssinosis’, there were significant associations with smoking (table 1). In the adjusted model that included job title, job status and working hours, byssinosis was not significantly associated with the duration of employment in the textile industry, although the ORs increased in the upper two quartiles. There was no significant association with type of current work (weaving vs spinning), although higher odds of byssinosis among spinners, as was evident for chronic bronchitis.

The overall mean (±SD) prebronchodilator values for lung function indices were as follows: FEV_1_: 3.004 (±0.581) L; FVC: 3.675 (±0.643) L; FEV_1_/FVC: 0.82 (±0.07). The reciprocal postbronchodilator values were: FEV_1_: 3.061 (±0.588) L; FVC: 3.667 (±0.641) L; FEV_1_/FVC: 0.83 (±0.07). On prebronchodilator tests, percentage-predicted values using NHANES-III reference equations were (mean±SD): FEV_1_: 77% (±13), FVC: 78% (±12) and ratio 100% (±8); approximately half (58%) of the participants had values of FEV_1_ and FVC below 80%; in contrast, only 2% (n=37) had a reduced FEV_1_/FVC ratio. The prevalence of ‘CAO’ was low (4%; n=74). A BDR in FEV_1_ was evident in 15% (n=253) of employees; in a sensitivity analysis considering an alternate definition of BDR (≥10% improvement in predicted percentage of FEV_1_)^21^ the prevalence was 7.5% (n=128). There were no important changes in the adjusted models reported below, using the alternate definition of BDR.

After adjusting for potential confounders, we found a significantly lower FEV_1_ among those with the longest duration of employment in the textile industry (with a clear trend across its quartiles), for those working in spinning sections (−0.147 L; 95% CI −0.202 to –0.092), and for those working shifts longer than 8 hours (−0.065 L; 95% CI −0.124 to –0.007) (see table 2). Findings in the adjusted FVC model were similar (spinning section: −0.136 L; 95% CI −0.198 to –0.007; more than 8 hours: −0.067 L; 95% CI −0.133 to –0.001), although there was no significant association with duration of employment in the textile industry. In contrast to FEV_1_ and FVC models, cleaners had decreased values (−1.6%; 95% CI −3.1% to –0.1%) for the FEV_1_/FVC ratio; but like these, spinners had decreased values for ratio (−1.2%; 95% CI −1.9% to –0.4%). We did not find an association with lung function for any of the other job titles. In the multivariable models for lung function outcomes (BDR, BDR with symptoms and/or asthma, and CAO) adjusting for age, pack-years of smoking, and job title, we did not find an increased risk with increased duration of employment, except for CAO. Spinners were at more than twofold increased risk of CAO compared with weavers. There was no clear relationship between BDR and byssinosis; of the 57 men with WHO symptoms-based byssinosis, 12 (21%) had a BDR, which was also 21% for Schilling’s (14 of 66) (p>0.05).

**Table 2:** Univariable and multivariable linear and logistic regression analyses of factors associated with spirometric outcomes (n=1721)

Variables	FEV_1_ (L)		FVC (L)		FEV_1_/FVC (%)		CAO (n=74; 4%)		BDR with symptoms† (n=174; 10%)	
	Unadjusted β (95% CI)	Adjusted β (95% CI)	Unadjusted β (95% CI)	Adjusted β (95% CI)	Unadjusted β (95% CI)	Adjusted β (95% CI)	OR (95% CI)	AOR (95% CI)	OR (95% CI)	AOR (95% CI)
Age (years)‡										
Q1 (18–24)	Reference	Reference	Reference	Reference	Reference	Reference	1.00	–	1.00	1.00
Q2 (25–30)	−0.126 (−0.196 to 0.055)	−0.113 (−0.179 to 0.046)	−0.041 (−0.122, 0.04)	−0.032 (−0.107, 0.043)	−2 (−3 to 2)	−2 (−3 to 1)	1.26 (0.59 to 2.69)	–	0.88 (0.57 to 1.36)	0.81 (0.51 to 1.28)
Q3 (31–37)	−0.264 (−0.335 to 0.192)	−0.227 (−0.304 to 0.150)	−0.117 (−0.2 to 0.034)	−0.101 (−0.187 to 0.014)	−5 (−5 to 4)	−4 (−5 to 3)	1.49 (0.71 to 3.15)	–	1.01 (0.65 to 1.55)	0.76 (0.45 to 1.28)
Q4 (38–71)	−0.592 (−0.663 to 0.522)	−0.534 (−0.618 to 0.449)	−0.45 (−0.531 to 0.368)	−0.425 (−0.520 to 0.330)	−7 (−8 to 6)	−6 (−7 to 5)	2.64 (1.36 to 5.13)	–	0.89 (0.57 to 1.37)	0.58 (0.32 to 1.05)
Height (cm)	0.035 (0.03 to 0.039)	0.036 (0.032, 0.04)	0.046 (0.041 to 0.05)	0.047 (0.043 to 0.051)	−0 (−0 to 0)	−0 (−0 to 0)	1.02 (0.99 to 1.06)	–	1.04 (1.01 to 1.07)	1.04 (1.01 to 1.07)
Pack-years of smoking										
None	Reference	Reference	Reference	Reference	Reference	Reference	1.00	1.00	1.00	1.00
<3.5	0.043 (−0.04 to 0.125)	0.064 (−0.006 to 0.134)	0.114 (0.022 to 0.206)	0.12 (0.041 to 0.199)	−1 (−2 to 0)	−1 (−2 to 0)	0.81 (0.34 to 1.91)	0.86 (0.36 to 2.07)	1.62 (1.05 to 2.52)	1.65 (1.06 to 2.57)
≥3.5	−0.329 (−0.411 to 0.248)	−0.143 (−0.216 to 0.069)	−0.203 (−0.293 to 0.112)	−0.066 (−0.149 to 0.017)	−5 (−6 to 4)	−3 (−4 to 2)	3.37 (2.00 to 5.66)	2.72 (1.54 to 4.81)	1.80 (1.19 to 2.73)	1.88 (1.19 to 2.96)
Duration of employment in textile industry (years)‡										
Q1 (0–4)	Reference	Reference	Reference	Reference	Reference	Reference	1.00	1.00	1.00	1.00
Q2 (5–9)	−0.07 (−0.146 to 0.006)	−0.027 (−0.098 to 0.044)	−0.048 (−0.134 to 0.037)	−0.025 (−0.105 to 0.055)	−1 (−2 to 0)	−0 (−1 to 1)	0.82 (0.37 to 1.83)	0.99 (0.42 to 2.31)	1.03 (0.65 to 1.63)	1.08 (0.67 to 1.74)
Q3 (10–16)	−0.185 (−0.258 to 0.112)	−0.07 (−0.147 to 0.007)	−0.115 (−0.198 to 0.032)	−0.055 (−0.142 to 0.032)	−2 (−3 to 2)	−1 (−2 to 0)	1.36 (0.68 to 2.73)	1.61 (0.74 to 3.53)	1.06 (0.68 to 1.65)	1.14 (0.69 to 1.89)
Q4 (17–46)	−0.433 (−0.508 to 0.357)	−0.112 (−0.206 to 0.018)	−0.315 (−0.4 to 0.23)	−0.087 (−0.193 to 0.019)	−5 (−6 to 4)	−1 (−2 to 0)	2.26 (1.17 to 4.33)	2.34 (1.04 to 5.26)	1.22 (0.79 to 1.91)	1.35 (0.74 to 2.47)
Section of mill										
Weaving	Reference	Reference	Reference	Reference	Reference	Reference	1.00	1.00	1.00	1.00
Spinning	−0.109 (−0.163 to 0.054)	−0.147 (−0.202 to 0.092)	−0.126 (−0.187 to 0.066)	−0.137 (−0.199 to 0.075)	−0 (−1 to 0)	−1 (−2 to 0)	2.61 (1.58 to 4.32)	2.67 (1.60 to 4.46)	1.09 (0.80 to 1.49)	1.09 (0.80 to 1.49)
Job title										
Supervisor	Reference	Reference	Reference	Reference	Reference	Reference	1.00	1.00	1.00	–
Technician	0.096 (−0.031 to 0.223)	0.018 (−0.089 to 0.125)	0.084 (−0.056 to 0.224)	0.057 (−0.064 to 0.178)	1 (−1 to 3)	−1 (−2 to 1)	0.78 (0.28, 2.16)	0.99 (0.35 to 2.81)	0.89 (0.44 to 1.79)	–
Machine operator	0.094 (−0.012 to 0.22)	0.02 (−0.072 to 0.112)	0.065 (−0.052 to 0.182)	0.072 (−0.031 to 0.176)	1 (0, 3)	−1 (−2 to 0)	0.74 (0.32 to 1.68)	1.24 (0.53 to 2.93)	0.84 (0.47 to 1.49)	–
Cleaner	0.094 (−0.023 to 0.21)	−0.026 (−0.133 to 0.082)	0.036 (−0.093 to 0.165)	0.038 (−0.084 to 0.159)	2 (0 to 3)	−2 (−3 to 0)	0.89 (0.36 to 2.20)	1.96 (0.70, 5.49)	0.90 (0.48 to 1.70)	–
Working hours										
≤8	Reference	Reference	Reference	Reference	Reference	Reference	1.00	–	1.00	–
>8	−0.003 (−0.062 to 0.057)	−0.065 (−0.124 to 0.006)	0.009 (−0.056 to 0.075)	−0.067 (−0.133 to 0.001)	−0 (−1 to 0)	−0 (−1 to 0)	0.82 (0.49 to 1.39)	–	1.07 (0.76 to 1.50)	–

All variables in the multivariable models are mutually adjusted; blank cells represent covariables that were dropped in the process of stepwise model building.

*Postbronchodilator FEV_1_/FVC ratio below LLN.

†BDR and presence of composite respiratory variable (includes chronic cough, chronic phlegm, increased cough and phlegm during last 3 years, wheezing, chest tightness current, breathlessness grades 1, 2 or 3) and/or self-reported asthma.

‡Continuous variables were converted into quartiles; Q1–Q4 correspond to the cut-off values for each quartile.

AOR, adjusted OR; BDR, bronchodilator response; CAO, chronic airflow obstruction; FEV_1_, forced expiratory volume in first second; FVC, forced vital capacity; LLN, lower limit of normality.

In a stratified analysis according to spinning or weaving type of mill, we did not observe large differences in sociodemographic or workplace characteristics, or the main respiratory outcomes measured, except for a higher frequency of CAO among those in spinning (6.4%) compared with weaving mills (2.5%). The regression analyses using this stratified approach showed largely similar findings across the two groups (online supplemental tables 1A,1B and 2A,2B).

10.1136/oemed-2022-108533.supp1 Supplementary data

**Figure 1:**
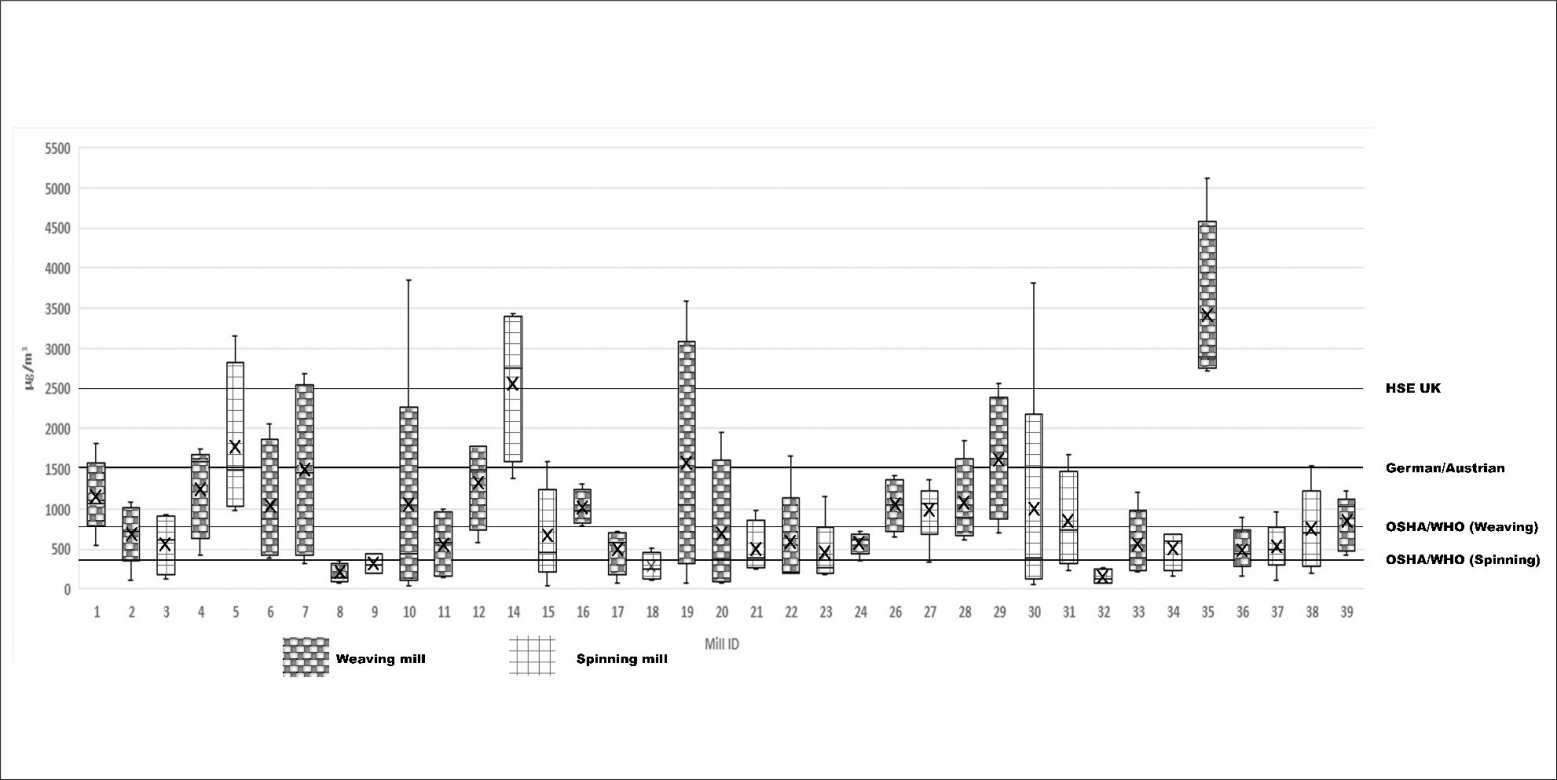
personal dust exposure levels at textile mills compared with international standards (n=37 mills). UK standards are based on ‘personal’ cotton dust measurements while others are based on ‘area’ measurements. HSE, Health & Safety Executive; OSHA, Occupational Safety & Health Administration; UK, United Kingdom; WHO, World Health Organisation.

The overall geometric mean (±GSD) personal dust level was 610 µg/m3, levels were higher among workers from the smaller mills (837 µg/m^3^±3) and among those from mills following a longer (12-hour) working shift (709 µg/m^3^±3). With respect to job titles, we found higher exposures among cleaners (752 µg/m^3^±3) and machine operators (649 µg/m^3^±3); this pattern was similar when we stratified data according to the spinning and weaving sections. We did not see a large difference in dust levels between spinning and weaving mills; in spinning mills, we found the highest exposures among those working in the subsection ‘blow room’ (929 µg/m^3^±2). In the weaving mills, we found personal dust exposures to be higher among those working on power-looms (902 µg/m^3^±3) or air-jet machines (716 µg/m^3^±3), compared with shuttle-less looms (491 µg/m^3^±3). A few instances aside, dust levels were below the UK cotton dust standard of 2500 µg/m^3^ but not the lower standards set by Occupational Safety & Health Administration (OSHA)/WHO (see figure 1).

## Discussion

In a large survey of respiratory health in contemporary textile manufacturing, we found a high prevalence of respiratory symptoms, 56% of participants having at least one symptom and 43% reporting shortness of breath (grade 1 or more). Duration of employment in the textile industry was identified as a key factor associated with most respiratory symptoms, independently of other variables, and with a clear gradient of increasing risk across its quartiles. A similar relationship was identified for spirometric outcomes indicative of obstructive lung disease. These findings are consistent with cumulative exposure to cotton textile dust being causal in the development of chronic respiratory symptoms and airflow obstruction—irrespective of the likely overlap between chronic byssinosis, asthma and chronic obstructive pulmonary disease (COPD).

Chest tightness was reported by 15% of participants, although in only 4% was the pattern of symptoms consistent with standard definitions of byssinosis. This figure (4%) is lower than the lowest prevalence we reported (6%) in a recent systematic review of the contemporary prevalence of byssinosis^3^; and lower than the Shanghai textile workers study (8%).^22^ While there was, in the present survey, a clear relationship between chest tightness and duration of employment in the textile industry, this was less obviously the case with byssinosis where although the adjusted ORs were higher with increased length of employment, none of them was statistically significantly raised. The pattern with job title was similar. This apparent anomaly between ‘chest tightness’ and byssinosis’ has a number of potential explanations. The first is limited statistical power due to the small number of byssinosis cases (n=60). A second is that the survival pressures of simple ‘chest tightness’ and byssinosis may be different. If the latter is a more severe condition, then employees with it may move away from exposed jobs at a higher rate. This may be consistent with the interesting observation that the ORs for many symptoms decreased with increasing age, independently of duration of employment in the textile industry.

The low prevalence of byssinosis we observed may, further, reflect relatively low levels of dust in the study mills since these were generally lower than those reported from other LMICs using the same gravimetric method for personal monitoring.^23–25^ Our estimates are similar to those reported by studies conducted among Lancashire textile workers in the UK at the end of 20th century.^1 26^ Both the low prevalence of byssinosis and low dust levels may reflect some undocumented improvements in Karachi mills since there appears to be a gradual decline in disease prevalence during the last decade.^11 27 28^ Several studies, both in LMICs^29 30^ and high-income countries,^1 31^ report potential links between the modernisation of machinery and ventilation systems in textile mills and decreased dust levels.

During the questionnaire-based interviews, it was clear that textile workers frequently had difficulty in understanding the byssinosis-related questions, which have a complex phrasing. Participants found it hard to relate symptoms of chest tightness to different days of the workweek in terms of ‘some’ or ‘most’ of the initial days back at work after a holiday. Variations in the prevalence of byssinosis (8%–38%) between similar recent surveys,^3^ may reflect similar differences in operational definitions and the difficulty faced by study participants in comprehending questions used to relate byssinosis with workplace exposure. Similar concerns have been expressed by authors from France^32^ and India.^33^

As previously reported,^24^ pack-years of smoking was associated with an increased likelihood of respiratory symptoms in our study, although there was no clear trend observed across the categories; perhaps due to the low cut-off we used to define the categories (3.5 pack-years). In this study, there was no clear association between respiratory symptoms and the type of mill (spinning vs weaving) although we did find an increased likelihood of abnormal spirometric outcomes among spinners.

We believe that this is among the largest surveys of respiratory health of textile workers. Other strengths include a high response rate, good quality spirometry and a substantial number of personal dust measurements. The fact that we chose a convenience sample of workers who were available at the time of the survey may have introduced a selection bias, leading to an underestimation of the respiratory health effects of cotton dust exposure. Other limitations include a potential survival effect—reflected by decreasing ORs for respiratory symptoms with increasing age—as well as the difficulty faced by our study participants in understanding questions for screening byssinosis.

Based on our experience, it seems that using either Schilling’s, or the WHO, symptoms-based criteria for identifying byssinosis among textile workers in LMICs may remain challenging because of the complex phrasing of questions and difficulty in differentiating between grades. While such a classification may be relevant in a clinical practice, it may prove less useful for epidemiological surveys and may lead to misclassification. It follows that simpler assessment methods would better identify byssinosis in contemporary textile manufacturing in LMICs. We propose deliberations on a simpler criterion of chest tightness in relation to work for use in workplace surveys; for the assessment of more advanced disease, we have argued elsewhere^28^ that a reduction in FEV_1_/FVC ratio rather than FEV_1_ is a more standardised approach for making comparisons across countries.

## Conclusion

In a large survey on health of textile workers, we found a high prevalence of respiratory symptoms but a low prevalence of byssinosis. Most respiratory outcomes, including those based on spirometry were associated with duration of employment in textile industry. We have discussed possible reasons for the low prevalence of byssinosis and the difficulty in using current, standard definitions.

## Data Availability

Data are available on reasonable request. Deidentified participant data may be acquired on reasonable request from the corresponding author (AAN).
