# Supplementary material for: Byssinosis and lung health among cotton textile workers: baseline findings of the MultiTex trial in Karachi, Pakistan
Source: Occup Environ Med. 2023 Jan 30;80(3):129–36. doi: 10.1136/oemed-2022-108533 (PMC9985716; doi:10.1136/oemed-2022-108533)
Supplement: Supplementary data [file oemed-2022-108533supp001.pdf]

Supplementary Table 1a Univariable and multivariable logistic regression analyses of individual and workplace factors in association with respiratory symptoms and byssinosis among textile workers from spinning mills (n=1048)

**Variables**	**n (%)**	**Chronic cough** (n=136; 13%)		**Chronic bronchitis**[1] (n=86; 8%)		**Composite respiratory variable**[2] (n=564; 54%)		**Byssinosis**[3] (n=32; 3%)	
		**OR (95% CI)**	**AOR (95% CI)**	**OR (95% CI)**	**AOR (95% CI)**	**OR (95% CI)**	**AOR (95% CI)**	**OR (95% CI)**	**AOR (95% CI)**
Age (years)[4]	28 (23-35)[5]								
Q1 (18-24)	-	1.00	1.00	1.00	1.00	1.00	1.00	1.00	-
Q2 (25-30)	-	1.15 (0.70, 1.88)	0.95 (0.56, 1.61)	1.22 (0.66, 2.24)	1.02 (0.54, 1.93)	1.04 (0.76, 1.42)	0.89 (0.60, 1.30)	2.21 (0.85, 5.78)	-
Q3 (31-37)	-	1.54 (0.93, 2.57)	0.95 (0.61, 1.48)	1.56 (0.83, 2.92)	0.87 (0.48, 1.57)	1.05 (0.74, 1.48)	0.49 (0.31, 0.79)	2.51 (0.92, 6.84)	-
Q4 (38-71)	-	1.77 (1.08, 2.89)	0.94 (0.51, 1.75)	1.75 (0.96, 3.21)	0.92 (0.43, 1.96)	1.44 (1.02, 2.03)	0.60 (0.35, 1.03)	1.31 (0.41, 4.18)	-
BMI[6]									
normal	433 (50.7)	1.00	-	1.00	-	1.00	1.00	1.00	-
underweight (<18.5)	156 (18.3)	1.18 (0.72, 1.94)	-	0.85 (0.44, 1.62)	-	1.18 (0.81, 1.72)	1.14 (0.78, 1.68)	1.52 (0.59, 3.88)	-
overweight and obese (>23.9)	265 (31.0)	0.92 (0.59, 1.42)	-	1.01 (0.61, 1.69)	-	1.43 (1.04, 1.96)	1.39 (0.98, 1.99)	1.40 (0.62, 3.17)	-
Pack-years of smoking									
none	818 (78.0)	1.00	1.00	1.00	1.00	1.00	1.00	1.00	1.00
<3.5	101 (9.6)	1.97 (1.15, 3.37)	1.86 (1.07, 3.23)	2.57 (1.39, 4.76)	2.54 (1.35, 4.79)	2.07 (1.34, 3.20)	1.95 (1.19, 3.20)	2.66 (1.04, 6.81)	2.40 (0.91, 6.29)
≥3.5	129 (12.3)	1.92 (1.18, 3.13)	1.47 (0.86, 2.51)	2.54 (1.45, 4.46)	2.20 (1.18, 4.10)	3.32 (2.17, 5.09)	3.33 (1.98, 5.60)	2.41 (0.99, 5.86)	2.02 (0.78, 5.28)
Duration of employment in the textile industry (years)[4]	9.0 (4.0–15.0)[5]								

Q1 (0-4)	-	1.00	1.00	1.00	1.00	1.00	1.00	1.00	1.00
Q2 (5-9)	-	1.69 (0.98, 2.91)	1.83 (1.01, 3.31)	1.62 (0.83, 3.16)	1.81 (0.88, 3.74)	1.28 (0.92, 1.78)	1.35 (0.89, 2.05)	0.76 (0.21, 2.72)	0.85 (0.22, 3.23)
Q3 (10-16)	-	1.55 (0.90, 2.70)	1.65 (0.86, 3.16)	1.57 (0.80, 3.08)	1.64 (0.74, 3.61)	1.67 (1.19, 2.33)	1.81 (1.14, 2.86)	2.59 (0.97, 6.93)	2.76 (0.92, 8.28)
Q4 (17-46)	-	2.80 (1.65, 4.73)	3.15 (1.47, 6.77)	2.52 (1.32, 4.81)	2.56 (1.02, 6.42)	2.00 (1.40, 2.85)	2.47 (1.36, 4.48)	2.12 (0.74, 6.05)	2.31 (0.66, 8.06)
Job title									
supervisor	89 (8.5)	1.00	1.00	1.00	1.00	1.00	1.00	1.00	1.00
technician	164 (15.6)	1.10 (0.48, 2.08)	1.35 (0.63, 2.89)	0.94 (0.38, 2.35)	1.31 (0.51, 3.36)	1.05 (0.62, 1.77)	1.45 (0.76, 2.78)	1.09 (0.20, 6.06)	1.50 (0.26, 8.56)
machine operator	541 (51.6)	0.87 (0.46, 1.65)	1.29 (0.65, 2.57)	0.92 (0.42, 2.02)	1.30 (0.56, 3.01)	0.85 (0.54, 1.33)	1.18 (0.66, 2.13)	1.58 (0.36, 6.92)	2.23 (0.49, 10.16)
cleaner	254 (24.4)	0.75 (0.37, 1.52)	1.74 (0.77, 3.92)	0.82 (0.35, 1.94)	1.83 (0.68, 4.91)	0.77 (0.47, 1.25)	1.64 (0.83, 3.21)	1.23 (0.25, 6.05)	2.90 (0.51, 16.55)
Working hours									
≤8	852 (81.3)	1.00	1.00	1.00	-	1.00	-	1.00	-
>8	196 (18.7)	0.50 (0.29, 0.88)	0.54 (0.30, 0.95)	0.55 (0.28, 1.08)	-	0.41 (0.30, 0.57)	-	0.61 (0.21, 1.77)	-

OR: odds ratio; AOR: adjusted odds ratio; CI: confidence interval; BMI: body-mass index; IQR: inter-quartile range

[1]Those having both cough and phlegm for 2 years or more

[2]WHO symptoms-based criteria

[3]Includes participants reporting one or more of respiratory symptoms (chronic cough, chronic phlegm, increased cough and phlegm during last 3 years, wheezing, breathlessness grades 1, 2 or 3, and chest tightness)

[4]Continuous variables were converted into quartiles; Q1-Q4 correspond to the cut-off values for each quartile

[5]Median (IQR) reported

[6]n=854

All variables in the multivariable models are mutually adjusted; blank cells represent covariables that were dropped in the process of stepwise model building

Supplementary Table 1b Univariable and multivariable logistic regression analyses of individual and workplace factors in association with respiratory symptoms and byssinosis among textile workers from weaving mills (n=983)

Variables	n (%)	Chronic cough (n=131; 13%)		Chronic bronchitis[1] (n=72; 7%)		Composite respiratory variable[2] (n=581; 59%)		Byssinosis[3] (n=28; 3%)	
		OR (95% CI)	AOR (95% CI)	OR (95% CI)	AOR (95% CI)	OR (95% CI)	AOR (95% CI)	OR (95% CI)	AOR (95% CI)
Age (years)[4]	31 (25-39)[5]								
Q1 (18-24)	-	1.00	1.00	1.00	1.00	1.00	1.00	1.00	-
Q2 (25-30)	-	1.05 (0.60, 1.83)	0.87 (0.47, 1.61)	1.91 (0.85, 4.33)	1.15 (0.48, 2.79)	1.04 (0.72, 1.50)	0.74 (0.49, 1.13)	0.65 (0.22, 1.89)	-
Q3 (31-37)	-	1.40 (0.82, 2.37)	0.85 (0.44, 1.67)	2.39 (1.08, 5.25)	0.81 (0.31, 2.15)	1.21 (0.83, 1.75)	0.66 (0.41, 1.06)	0.73 (0.26, 2.05)	-
Q4 (38-71)	-	1.00 (0.58, 1.73)	0.45 (0.21, 0.96)	1.81 (0.81, 4.07)	0.39 (0.13, 1.18)	1.03 (0.72, 1.49)	0.48 (0.29, 0.80)	0.68 (0.24, 1.91)	-
BMI[6]									
normal	459 (47.8)	1.00	-	1.00	-	1.00	1.00	1.00	-
underweight (<18.5)	113 (11.8)	1.24 (0.68, 2.25)	-	1.17 (0.52, 2.65)	-	1.24 (0.82, 1.89)	1.13 (0.74, 1.75)	0.87 (0.24, 3.07)	-
overweight and obese (>23.9)	389 (40.5)	1.39 (0.94, 2.07)	-	1.57 (0.94, 2.62)	-	1.32 (1.00, 1.74)	1.27 (0.95, 1.71)	0.92 (0.41, 2.06)	-
Pack-years of smoking									
none	726 (73.9)	1.00	1.00	1.00	1.00	1.00	1.00	1.00	1.00
<3.5	138 (14.0)	1.90 (1.17, 3.09)	1.91 (1.16, 3.14)	2.15 (1.15, 4.02)	2.10 (1.11, 3.98)	1.76 (1.20, 2.59)	1.72 (1.16, 2.55)	2.42 (1.03, 5.68)	2.47 (1.04, 5.84)
≥3.5	119 (12.1)	2.29 (1.40, 3.75)	2.52 (1.47, 4.33)	3.14 (1.73, 5.70)	2.73 (1.43, 5.22)	2.44 (1.58, 3.79)	2.46 (1.53, 3.95)	0.67 (0.15, 2.94)	0.64 (0.14, 2.91)
Duration of employment in the textile industry (years)[4]	10.0 (5.0–17.0)[5]								

Q1 (0-4)	-	1.00	1.00	1.00	1.00	1.00	1.00	1.00	1.00
Q2 (5-9)	-	0.95 (0.53, 1.72)	0.94 (0.50, 1.78)	2.52 (0.95, 6.68)	2.34 (0.83, 6.56)	1.60 (1.11, 2.32)	1.72 (1.14, 2.59)	0.89 (0.29, 2.68)	0.79 (0.25, 2.45)
Q3 (10-16)	-	1.37 (0.81, 2.33)	1.49 (0.76, 2.92)	3.41 (1.36, 8.57)	3.54 (1.20, 10.41)	1.70 (1.19, 2.42)	1.83 (1.16, 2.88)	0.87 (0.30, 2.53)	0.75 (0.25, 2.31)
Q4 (17-46)	-	1.62 (0.96, 2.74)	2.30 (1.05, 5.07)	5.20 (2.12, 12.74)	7.41 (2.22, 24.67)	1.95 (1.35, 2.80)	2.19 (1.28, 3.76)	1.08 (0.38, 3.02)	0.98 (0.32, 3.05)
Job title									
supervisor	65 (6.6)	1.00	1.00	1.00	1.00	1.00	1.00	1.00	1.00
technician	94 (9.6)	1.27 (0.41, 3.98)	1.67 (0.52, 5.42)	1.04 (0.28, 3.84)	1.46 (0.38, 5.60)	1.03 (0.54, 1.99)	1.14 (0.58, 2.28)	0.68 (0.09, 4.99)	0.63 (0.08, 4.74)
machine operator	642 (65.3)	2.14 (0.84, 5.45)	3.20 (1.21, 8.51)	1.43 (0.50, 4.08)	2.18 (0.74, 6.47)	0.91 (0.53, 1.54)	1.10 (0.63, 1.91)	1.01 (0.23, 4.43)	1.03 (0.23, 4.68)
cleaner	182 (18.5)	1.48 (0.53, 4.12)	2.76 (0.92, 8.32)	0.61 (0.17, 2.16)	1.80 (0.46, 7.01)	0.57 (0.32, 1.02)	0.92 (0.48, 1.74)	0.71 (0.13, 3.96)	0.67 (0.11, 4.21)
Working hours									
≤8	446 (45.4)	1.00	1.00	1.00	-	1.00	-	1.00	-
>8	537 (54.6)	0.82 (0.57, 1.19)	0.71 (0.48, 1.05)	0.92 (0.57, 1.49)	-	0.91 (0.71, 1.18)	-	0.96 (0.45, 2.03)	-

OR: odds ratio; AOR: adjusted odds ratio; CI: confidence interval; BMI: body-mass index; IQR: inter-quartile range

[1]Those having both cough and phlegm for 2 years or more

[2]WHO symptoms-based criteria

[3]Includes participants reporting one or more of respiratory symptoms (chronic cough, chronic phlegm, increased cough and phlegm during last 3 years, wheezing, breathlessness grades 1, 2 or 3, and chest tightness)

[4]Continuous variables were converted into quartiles; Q1-Q4 correspond to the cut-off values for each quartile

[5]Median (IQR) reported

[6]n=961

All variables in the multivariable models are mutually adjusted; blank cells represent covariables that were dropped in the process of stepwise model building

Supplementary Table 2a Univariable and multivariable linear and logistic regression analyses of factors associated with spirometric outcomes among textile workers from spinning mills (n=805)

Variables	FEV_1 (ml)		FVC (ml)		FEV_1/FVC (%)		CAO[1] (n=51; 6%)		BDR with symptoms[2] (n=85; 11%)	
	**Unadjusted β (95% CI)**	**Adjusted β (95% CI)**	**Unadjusted β (95% CI)**	**Adjusted β (95% CI)**	**Unadjusted β (95% CI)**	**Adjusted β (95% CI)**	**OR (95% CI)**	**AOR (95% CI)**	**OR (95% CI)**	**AOR (95% CI)**
Age (years)[3]										
Q1 (18-24)	Reference	Reference	Reference	Reference	Reference	Reference	1.00	-	1.00	1.00
Q2 (25-30)	-126 (-223, -29)	-89 (-184, - 6)	-37 (-145, 72)	-4 (-107, 100)	-2 (-4, -1)	-2 (-4, -1)	1.46 (0.61, 3.50)	-	1.12 (0.61, 2.03)	1.13 (0.60, 2.12)
Q3 (31-37)	-283 (-390, -176)	-188 (-303, -73)	-127 (-246, -8)	-64 (-189, 61)	-5 (-6, -4)	-4 (-5, -2)	2.01 (0.84, 4.86)	-	0.95 (0.48, 1.87)	0.75 (0.34, 1.69)
Q4 (38-71)	-637 (-740, -534)	-528 (-655, -401)	-487 (-602, -373)	-427 (-565, -289)	-7 (-9, -6)	-6 (-8, -4)	3.21 (1.45, 7.08)	-	1.44 (0.79, 2.61)	0.92 (0.40, 2.11)
Height (cm)	29 (23, 36)	33 (27, 39)	39 (32, 46)	43 (36, 49)	-0 (-0, 0)	-0 (-0, 0)	1.01 (0.97, 1.06)	-	1.03 (1.00, 1.07)	1.03 (0.99, 1.07)
Pack-years of smoking										
none	Reference	Reference	Reference	Reference	Reference	Reference	1.00	1.00	1.00	1.00
<3.5	-114 (-247, 19)	-49 (-166, 68)	-39 (-184, 105)	9 (-118, 136)	-2 (-4, -1)	-2 (-3, 0)	1.28 (0.48, 3.42)	1.29 (0.48, 3.48)	1.78 (0.91, 3.48)	1.88 (0.95, 3.72)
≥3.5	-321 (-438, -204)	-138 (-249, -28)	-159 (-286, -32)	-45 (-165, 75)	-6 (-8, -4)	-3 (-5, -2)	3.66 (1.94, 6.93)	2.98 (1.48, 5.99)	2.34 (1.33, 4.09)	2.32 (1.23, 4.38)
Duration of employment in textile industry (years)[3]										
Q1 (0-4)	Reference	Reference	Reference	Reference	Reference	Reference	1.00	1.00	1.00	1.00
Q2 (5-9)	-74 (-182, 34)	-48 (-155, 58)	-50 (-168, 69)	-50 (-166, 66)	-1 (-2, 1)	-0 (-2, 1)	1.07 (0.42, 2.75)	1.39 (0.50, 3.83)	1.02 (0.54,1.91)	0.98 (0.51, 1.87)
Q3 (10-16)	-172 (-278, -65)	-67 (-181, 47)	-99 (-215, 18)	-50 (-174, 74)	-2 (-4, -1)	-1 (-3, 1)	1.63 (0.69, 3.84)	1.99 (0.75, 5.24)	0.77 (0.40, 1.49)	0.71 (0.34, 1.46)
Q4 (17-46)	-488	-168	-348	-125	-6	-2	2.72	2.85	1.43	1.16

	(-599, -376)	(-312, -23)	(-470, -226)	(-282, 32)	(-7, -4)	(-4, 0)	(1.20, 6.18)	(1.03, 7.92)	(0.77, 2.63)	(0.50, 2.68)
Job title										
supervisor	Reference	Reference	Reference	Reference	Reference	Reference	1.00	1.00	1.00	-
technician	286	111	265	151	2	-0	0.75	1.08	1.02	-
	(114, 458)	(-42, 264)	(81, 449)	(-15, 318)	(-0, 5)	(-3, 2)	(0.23, 2.45)	(0.32, 3.66)	(0.41, 2.55)	
machine	204	50	181	128	2	-2	0.82	1.31	0.82	-
operator	(54, 352)	(-88, 187)	(22, 340)	(-21, 277)	(-0, 4)	(-4, 0)	(0.30, 2.22)	(0.46, 3.69)	(0.37, 1.84)	
cleaner	238 (76, 400)	-11 (-171, 150)	160 (-13, 333)	49 (-125, 224)	3 (1, 5)	-2 (-4, 0)	0.89 (0.36, 2.20)	2.45 (0.72, 8.35)	0.90 (0.38, 2.16)	-
Working hours										
≤8	Reference	Reference	Reference	Reference	Reference	Reference	1.00	-	1.00	-
>8	-54 (-239, 131)	-49 (-209, 109)	-85 (-282, 113)	-108 (-281, 65)	-0 (-3, 2)	0 (-2, 3)	1.64 (0.56, 4.81)	-	1.18 (0.45, 3.10)	-

FEV_1: forced expiratory volume in first second; FVC: forced vital capacity; CAO: chronic airflow obstruction; BDR: bronchodilator reversibility; OR: odds ratio; AOR: adjusted odds ratio; CI: confidence interval; LLN: lower limit of normality

[1]Post-bronchodilator FEV_1/FVC ratio below LLN; (n=803)

[2] BDR and presence of composite respiratory variable (includes chronic cough, chronic phlegm, increased cough and phlegm during last 3 years, wheezing, chest tightness current, breathlessness grades 1, 2 or 3), and/or self-reported asthma

[3]Continuous variables were converted into quartiles; Q1-Q4 correspond to the cut-off values for each quartile

All variables in the multivariable models are mutually adjusted; blank cells represent covariables that were dropped in the process of stepwise model building

Supplementary Table 2b Univariable and multivariable linear and logistic regression analyses of factors associated with spirometric outcomes among textile workers from weaving mills (n=916)

**Variables**	**FEV_1 (ml)**		**FVC (ml)**		**FEV_1/FVC (%)**		**CAO[1]** (n=23; 2%)		**BDR with symptoms[2]** (n=89; 10%)	
	**Unadjusted β (95% CI)**	**Adjusted β (95% CI)**	**Unadjusted β (95% CI)**	**Adjusted β (95% CI)**	**Unadjusted β (95% CI)**	**Adjusted β (95% CI)**	**OR (95% CI)**	**AOR (95% CI)**	**OR (95% CI)**	**AOR (95% CI)**
Age (years)[3]										
Q1 (18-24)	Reference	Reference	Reference	Reference	Reference	Reference	1.00	-	1.00	1.00
Q2 (25-30)	-156 (-256, -55)	-141 (-236, -46)	-78 (-198, 41.40)	-64 (-175, 47)	-3 (-4 - -1)	-2 (-4, -1)	1.19 (0.26, 5.37)	-	0.67 (0.36, 1.27)	0.54 (0.27, 1.08)
Q3 (31-37)	-302 (-401, -203)	-259 (-365, -153)	-164 (-282, -46)	-130 (-254, -7)	-4 (-6 - -3)	-4 (-5, -3)	1.39 (0.33, 5.91)	-	0.97 (0.54, 1.73)	0.60 (0.28, 1.27)
Q4 (38-71)	-609 (-707, -512)	-533 (-648, -418)	-475 (-591, -359)	-417 (-552, -283)	-6 (-8 - -5)	-6 (-7 -4)	2.94 (0.81, 10.68)	-	0.55 (0.29, 1.05)	0.33 (0.14, 0.78)
Height (cm)	38 (33, 44)	39 (34, 44)	50 (44, 56)	51 (45 - 57)	-0 (-0 - 0)	-0 (-0, 0)	1.07 (1.00, 1.14)	1.07 (1.00, 1.15)	1.05 (1.02, 1.09)	1.05 (1.01, 1.09)
Pack-years of smoking										
none	Reference	Reference	Reference	Reference	Reference	Reference	1.00	1.00	1.00	1.00
<3.5	127 (23, 230)	139 (52, 226)	191 (72, 310)	190 (88 - 291)	-1 (-2, -0)	-0 (-2, 1)	0.33 (0.04, 2.52)	0.35 (0.05, 2.66)	1.52 (0.85, 2.69)	1.52 (0.85, 2.73)
≥3.5	-335 (-447, -224)	-141 (-239, -42)	-242 (-371, -114)	-84 (-199, 31)	-4 (-5, -3)	-2 (-3, -1)	2.86 (1.14, 7.17)	2.78 (1.07, 7.18)	1.33 (0.71, 2.52)	1.51 (0.76, 3.00)
Duration of employment in textile industry (years)[3]										
Q1 (0-4)	Reference	Reference	Reference	Reference	Reference	Reference	1.00	-	1.00	1.00
Q2 (5-9)	-68 (-173, 36)	-7 (-103, 88)	-50 (-171, 72)	-5 (-117, 106)	-1 (-2, 1)	0 (-1, 1)	0.41 (0.08, 2.14)	-	1.04 (0.54, 2.03)	1.25 (0.62, 2.52)
Q3 (10-16)	-204 (-305, -104)	-76 (-181, 29)	-140 (-256, -23)	-69 (-192, 54)	-2 (-4, -1)	-0 (-2, 1)	1.05 (0.32, 3.49)	-	1.38 (0.75, 2.54)	1.85 (0.88, 3.89)

Q4 (17-46)	-404 (-505, -302)	-83 (-208, 41)	-306 (-424, -187)	-71 (-216, 74)	-4 (-5, -3)	-1 (-2, 1)	1.88 (0.63, 5.60)	-	1.08 (0.57, 2.05)	1.75 (0.72, 4.27)
Job title										
supervisor	Reference	Reference	Reference	Reference	Reference	Reference	1.00	1.00	1.00	-
technician	-119 (-306, 67)	-146 (-300, 8)	-117 (-330, 96)	-109 (-289, 72)	-0 (-2, 2)	-1 (-3, 1)	0.71 (0.10, 5.21)	0.67 (0.09, 5.03)	0.70 (0.23, 2.10)	-
machine operator	-55 (-205, 94)	-48 (-174, 77)	-92 (-263, 78)	-21 (-168, 126)	1 (-1, 2)	-1 (-2, 1)	0.78 (0.17, 3.47)	0.94 (0.20, 4.42)	0.85 (0.37, 1.95)	-
cleaner	-67 (-234, 100)	-86 (-234, 62)	-102 (-292, 89)	-18 (-190, 155)	1 (-1, 3)	-2 (-4, 0)	0.74 (0.13, 4.15)	1.16 (0.20, 6.87)	0.90 (0.35, 2.28)	-
Working hours										
≤8	Reference	Reference	Reference	Reference	Reference	Reference	1.00	1.00	1.00	-
>8	-95 (-169, -21)	-57 (-118, 5)	-86 (-171, -2)	-44 (-116, 28)	-1 (-2, 0)	-0 (-1, 0)	2.00 (0.82, 4.92)	1.82 (0.73, 4.58)	1.18 (0.76, 1.83)	-

FEV_1: forced expiratory volume in first second; FVC: forced vital capacity; CAO: chronic airflow obstruction; BDR: bronchodilator reversibility; OR: odds ratio; AOR: adjusted odds ratio; CI: confidence interval; LLN: lower limit of normality

[1]Post-bronchodilator FEV_1/FVC ratio below LLN; (n=909)

[2] BDR and presence of composite respiratory variable (includes chronic cough, chronic phlegm, increased cough and phlegm during last 3 years, wheezing, chest tightness current, breathlessness grades 1, 2 or 3), and/or self-reported asthma

[3]Continuous variables were converted into quartiles; Q1-Q4 correspond to the cut-off values for each quartile

All variables in the multivariable models are mutually adjusted; blank cells represent covariables that were dropped in the process of stepwise model building
